# Crystallography, in Silico Studies, and In Vitro Antifungal Studies of 2,4,5 Trisubstituted 1,2,3-Triazole Analogues

**DOI:** 10.3390/antibiotics9060350

**Published:** 2020-06-20

**Authors:** Katharigatta N. Venugopala, Mohammed A. Khedr, Yarabahally R. Girish, Subhrajyoti Bhandary, Deepak Chopra, Mohamed A. Morsy, Bandar E. Aldhubiab, Pran Kishore Deb, Mahesh Attimarad, Anroop B. Nair, Nagaraja Sreeharsha, Rashmi V, Mahmoud Kandeel, Sabah H. Akrawi, Madhusudana Reddy M B, Sheena Shashikanth, Osama I. Alwassil, Viresh Mohanlall

**Affiliations:** 1 Department of Pharmaceutical Sciences, College of Clinical Pharmacy, King Faisal University, Al-Ahsa 31982, Saudi Arabia; mmohammed@kfu.edu.sa (M.A.K.); momorsy@kfu.edu.sa (M.A.M.); baldhubiab@kfu.edu.sa (B.E.A.); mattimarad@kfu.edu.sa (M.A.); anair@kfu.edu.sa (A.B.N.); sharsha@kfu.edu.sa (N.S.); sakrawi@kfu.edu.sa (S.H.A.); 2 Department of Biotechnology and Food Technology, Durban University of Technology, Durban 4001, South Africa; vireshm@dut.ac.za; 3 Department of Pharmaceutical Chemistry, Faculty of Pharmacy, Helwan University, Ein Helwan, Cairo 11795, Egypt; 4 ACU-Center for Research and Innovations, BGSIT Campus, Adichunchanagiri University, B.G. Nagara, Mandya District 571448, India; girish.yr@gmail.com; 5 Department of Chemistry, Indian Institute of Science Education and Research Bhopal, Bhopal By-pass Road, Bhauri, Bhopal 462066, India; bhandarysj@gmail.com (S.B.); dchopra@iiserb.ac.in (D.C.); 6 Department of Pharmacology, Faculty of Medicine, Minia University, El-Minia 61511, Egypt; 7 Faculty of Pharmacy, Philadelphia University, Amman 19392, Jordan; prankishore1@gmail.com; 8 Department of Pharmaceutics, Vidya Siri College of Pharmacy, Off Sarjapura Road, Bengaluru 560035, India; 9 Department of Public health Medicine, University of KwaZulu-Natal, Howard College Campus, Durban 4041, South Africa; rashmivenugopala@gmail.com; 10 Department of Biomedical Sciences, College of Veterinary Medicine, King Faisal University, Al-Ahsa 31982, Saudi Arabia; mkandeel@kfu.edu.sa; 11 Department of Pharmacology, Faculty of Veterinary Medicine, Kafrelsheikh University, Kafrelsheikh 33516, Egypt; 12 Department of Chemistry, School of Applied Sciences, REVA University, Bangalore 560064, India; mbmsreddy@gmail.com; 13 Department of Studies in Organic Chemistry, University of Mysore, Manasagangotri, Mysore 570006, India; shashikanth@chemistry.uni-mysore.ac.in; 14 Department of Pharmaceutical Sciences, College of Pharmacy, King Saud bin Abdulaziz University for Health Sciences, Riyadh 11481, Saudi Arabia; wassilo@ksau-hs.edu.sa

**Keywords:** 2,4,5 trisubstituted 1,2,3-triazoles, antifungal activity, single-crystal X-ray diffraction, minimum inhibitory concentration, molecular docking, dynamic studies

## Abstract

A series of 2,4,5 trisubstituted-1,2,3-triazole analogues have been screened for their antifungal activity against five fungal strains, *Candida parapsilosis*, *Candida albicans*, *Candida tropicalis*, *Aspergillus niger*, and *Trichophyton rubrum*, via a 3-[4,5-dimethylthiazol-2-yl]-2,5-diphenyltetrazolium bromide (MTT) microdilution assay. Compounds GKV10, GKV11, and GKV15 emerged as promising antifungal agents against all the fungal strains used in the current study. One of the highly active antifungal compounds, GKV10, was selected for a single-crystal X-ray diffraction analysis to unequivocally establish its molecular structure, conformation, and to understand the presence of different intermolecular interactions in its crystal lattice. A cooperative synergy of the C-H···O, C-H···N, C-H···S, C-H···π, and π···π intermolecular interactions was present in the crystal structure, which contributed towards the overall stabilization of the lattice. A molecular docking study was conducted for all the test compounds against *Candida albicans* lanosterol-14α-demethylase (pdb = 5 tzl). The binding stability of the highly promising antifungal test compound, GKV15, from the series was then evaluated by molecular dynamics studies.

## 1. Introduction

Fungi are organisms with similar properties to plants [1]. Many fungal infections may be superficial or systematic, such as candidiasis, aspergillosis, and dermatophytosis [2]. Some diseases can increase the incidence of fungal infections, such as cancer, human immunodeficiency virus, and the overuse of antibiotics [3,4]. Available antifungal drugs have many side effects and lack selectivity towards fungi, as they harm human cells, causing a hepatotoxic effect similar to azole antifungal agents [5]. In addition, they have poor solubility and bioavailability [6]. The development of selective and safe antifungal agents with enhanced bioavailability properties is a significant challenge for pharmaceutical companies. Lanosterol-14-α-demethylase is the enzyme responsible for the synthesis of ergosterol in fungi and is a specific component in the fungal cell membrane. The inhibition of ergosterol synthesis will cause death to the fungal cell. Hence, it is a potential selective target for antifungal azole agents [7]. In silico studies suggest that lanosterol-14-α-demethylase has a great affinity for azole moieties such as diazole, triazole, and tetrazole, to bind and coordinate to the heme found in the heme–porphyrin cofactor. To date, the reported use of triazole scaffolds in antifungal drugs has been limited. A triazole scaffold has many reported pharmacological properties, such as antiviral [8], hypoglycemic [9], anti-tubercular [10,11], antimicrobial [12,13] and antihypertensive properties [14]. In continuation to our effort to apply methods [15,16,17,18,19] for the development of pharmacologically active heterocyclic compounds [20,21,22,23,24,25,26,27,28,29,30,31,32,33,34,35], the current study aimed to investigate the in vitro antifungal activity of synthetic 1,3,4-trisubstituted-1,2,3-triazole analogues and to computationally evaluate their binding modes.

## 2. Results and Discussion

### 2.1. Chemistry

**Scheme 1:**
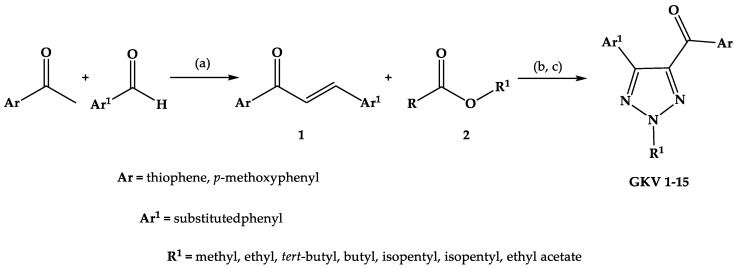
Synthesis of the title compounds GKV1–15. Reagents and conditions: (a) 6N NaOH, Ethanol, 5 h, r.t; (b) NaN_3_, ZrO_2_-Cu_2_-β-CD, DMF, 100 °C, stirred, 15 h; (c) 100 °C, stirred, 16 h.

**Figure 1:**
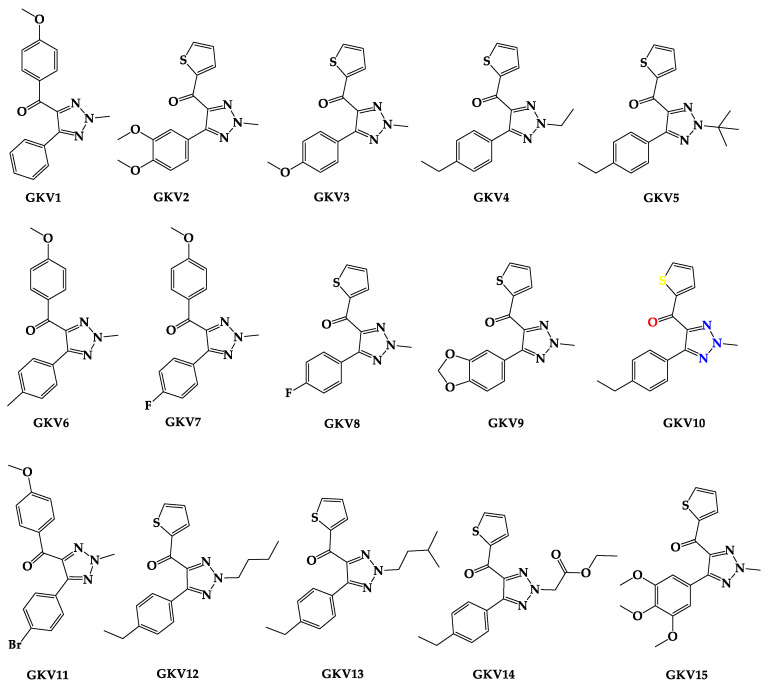
The molecular structures of 1,3,4-trisubstituted-1,2,3-triazole analogues (GKV1–15) studied for antifungal properties against *Candida parapsilosis*, *Candida albicans*, *Candida tropicalis*, *Aspergillus niger*, and *Trichophyton rubrum* by a 3-[4,5-dimethylthiazol-2-yl]-2,5-diphenyltetrazolium bromide (MTT) microdilution assay.

Synthesis of the title compounds GKV1–15 (Figure 1) was achieved by two-step chemical synthesis (Scheme 1). Characterization was achieved by FT-IR, NMR (^1^H and ^13^C), High-resolution mass spectrometry (HRMS), and selected compounds using the single-crystal X-ray method, as per an earlier study [36]. The yield of the compounds was in the range of 45–82%, and the purity of the compounds was over 99%. The title compound GKV3 was studied for single-crystal X-ray study analysis, and the intermolecular interactions were further evaluated through the molecular electrostatic potential map and Hirshfeld fingerprint analysis [37].

### 2.2. Crystallography

**Figure 2:**
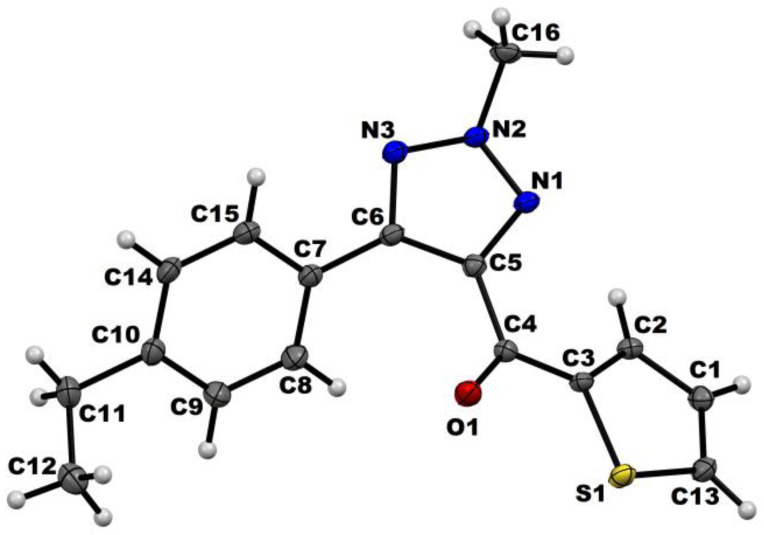
The Oak Ridge Thermal Ellipsoid Plot (ORTEP) of GKV10 drawn with 50% ellipsoidal probability.

**Figure 3:**
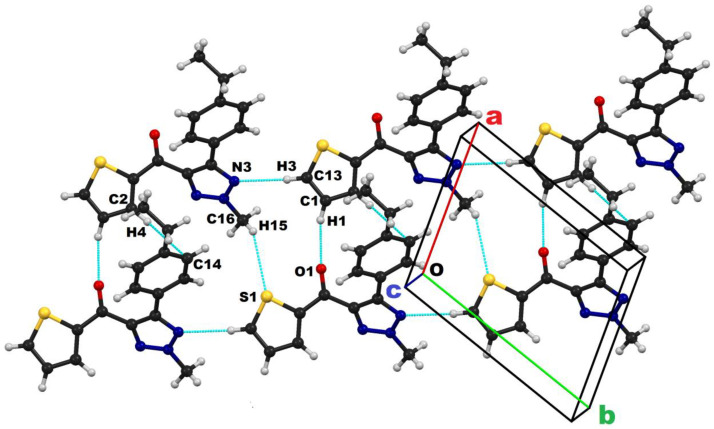
Packing network of molecules down the *ab* plane associated with C-H···N, C-H···O hydrogen bonds and C-H···S and C-H···π intermolecular contacts.

**Figure 4:**
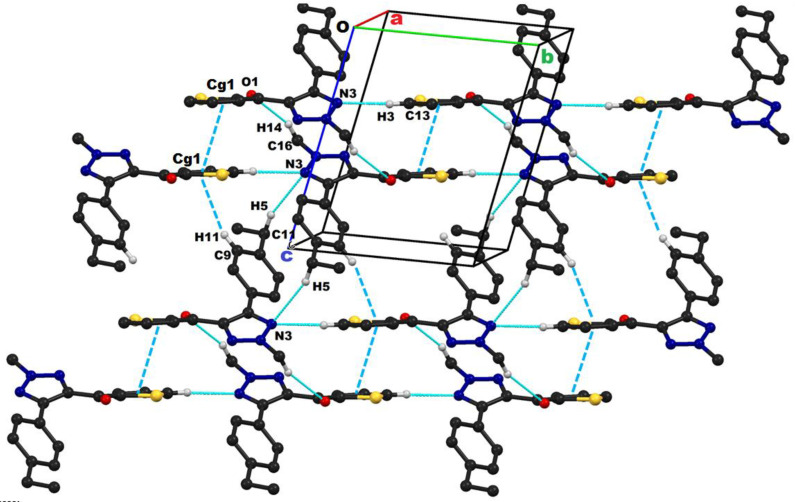
Packing of molecules down the *bc* plane associated with C-H···O, C-H···N, C-H···Cg1, and π···π stacking (Cg1···Cg1) interactions. All non-interacting hydrogen atoms were removed for clarity.

**Figure 5:**
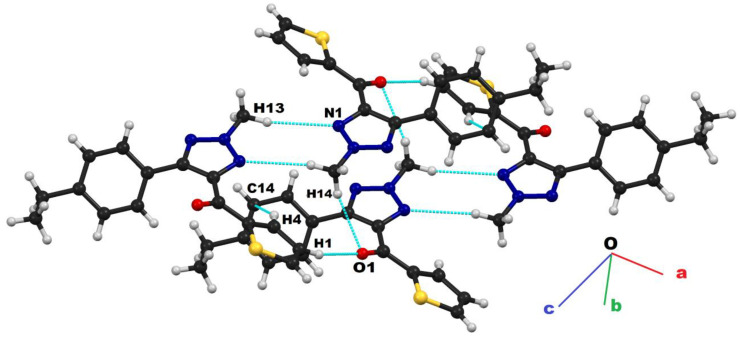
Centro-symmetric C-H···N dimers connected via C-H···O and C-H···π interactions.

The molecular structure of the molecule GKV10 in the crystal shows that it is conformationally flexible (Figure 2). The magnitudes of the torsion angles for C8-C7-C6-N3 and N1-C5-C4-O1 are 143.5° and 145.6°, respectively. Interestingly, the torsion angle for O1-C4-C3-C2 is 174.4°, indicating planarity in the solid-state structure. This could possibly result in increased delocalization of the electron density between the carbonyl groups and the thiophene ring. The packing of molecules in the crystal shows the presence of C-H···N hydrogen bonds (involving acidic H3 and N3) forming molecular chains (Figure 3). These parallel chains are translated and connected via C-H···O (involving H1 and O1), C-H···S (involving H15 and S1), and C-H···π (involving H4 and C14) intermolecular interactions. Centro-symmetrically related chains formed via C-H···N hydrogen bonds (involving acidic H3 and N3) are further stabilized via C-H···O interactions (involving H14 and O1) and π···π stacking interactions at a distance of 3.901(2) Å (Figure 4). Two such consecutive pairs of dimers are again centro-symmetrically related and connected via C-H···π (involving H11 and Cg1) and C-H···N (involving H5 and N3) (Figure 4). In addition, a different C-H···N dimer (involving H13 and N1) is present in the crystalline lattice, and a pair of such dimers are stabilized further via the already present C-H···O (involving H14 and O1) and C-H···π interactions (involving H4 and C14) (Figure 5).

### 2.3. Pharmacology

The in vitro antifungal minimum inhibitory concentration (MIC) was determined by using the two-fold dilution method (at 0.49, 0.98, 1.95, 3.9, 7.81, 15.63, 31.25, 62.5, and 125 µg/mL concentrations) and the results are presented in Table 1. Compounds GKV10, GKV11, and GKV15 were selective against *Candida* (*C.*) *tropicalis* (0.49 μg/mL). They showed similar activity against *C. parapsilosis* and *C. albicans*, as well (0.98 μg/mL). Compound GKV15 had higher activity against *Aspergillus* (*A.*) *niger* and *Trichophyton* (*T.*) *rubrum* (0.98 μg/mL). The promising results of these antifungal activities may require the optimization of highly active compounds in the future.

### 2.4. Molecular Docking

**Figure 6:**
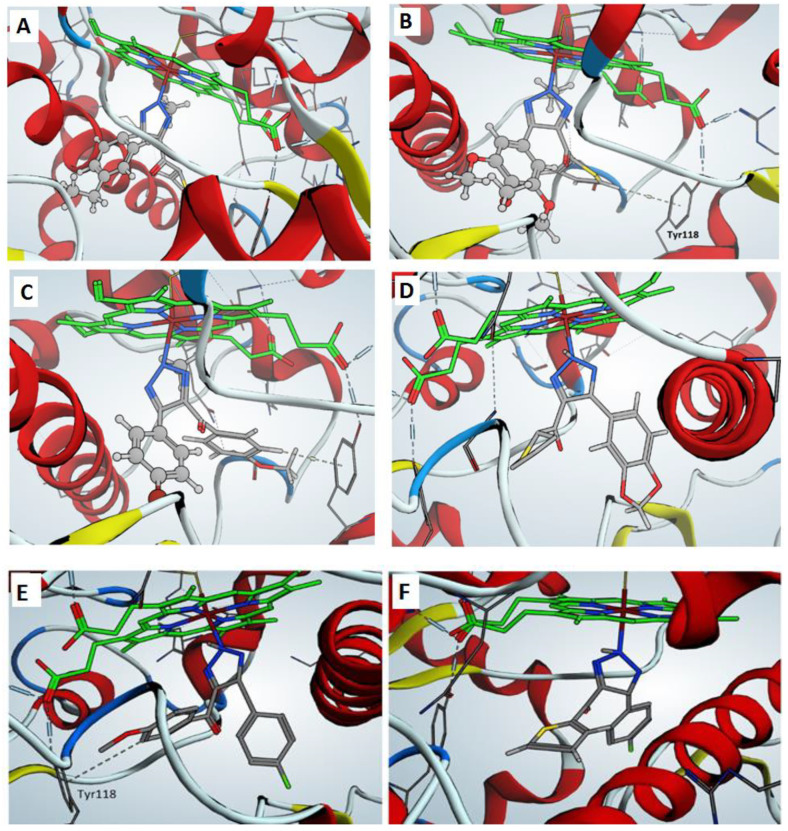
(**A**) Docking pose of the compound GKV10. (**B**) Docking pose of the compound GKV11. (**C**) Docking pose of the compound GKV15. (**D**) Docking pose of the compound GKV9. (**E**) Docking pose of the compound GKV7. (**F**) Docking pose of the compound GKV8.

The molecular docking study was performed to evaluate the binding orientation of the tested compounds within the active site of lanosterol-14-α-demethylase. We computed scores for the free energy of binding, affinity, clash score, and the root of the mean square deviation (RMSD). Compound GKV15 showed the top-ranked ΔG value (−24.75 kcal/mol), affinity (30.01), and was the lowest in RMSD (0.81 Ǻ) and clash score (2.25) (Table 2). Compounds GKV11, GKV10, GKV7, GKV8, and GKV9 also showed higher ΔG values of −24.54, −24.33, −23.98, −23.88, and −23.71 kcal/mol, respectively. The thiophene ring in the tested compounds contributed to the hydrophobicity required for the hydrophobic binding site. The triazole ring revealed two important roles: forming strong coordination with the heme of the heme–porphyrin complex (achieved in all of the tested compounds as shown in Figure 6) and maintaining the downward orientation of the two hydrophobic rings of the structure, which was also achieved successfully.

**Figure 7:**
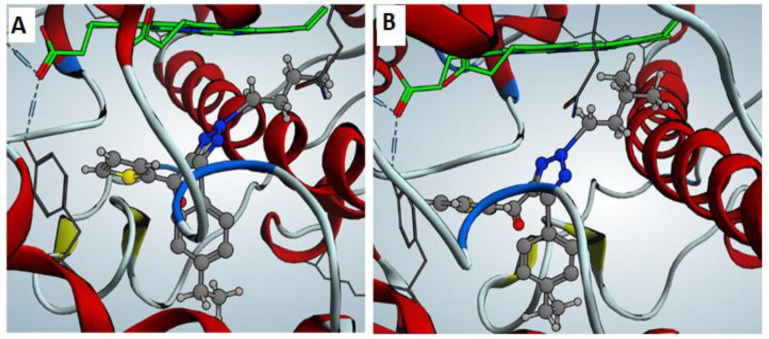
(**A**) Docking pose of compound GKV12. (**B**) Docking pose of compound GKV14.

The length of the alkyl side chain on the *N*-atom of the triazole ring affected the binding. Presence of methyl group in compounds like GKV1-3, GKV6-11, and 15 had an electron-donating effect that enhanced the electron cloud around the nitrogen atom of the triazole ring and potentiated its coordination with the heme. However, the branching as well as increase in the carbon chain length resulted in the increase in steric hindrance, thereby effecting the coordination with the heme which probably could be the reason for detrimental effect in the activity as evident in case of the compounds 4, 5, 12, 13 and 14 (Figure 7).

### 2.5. Molecular Dynamics

**Figure 8:**
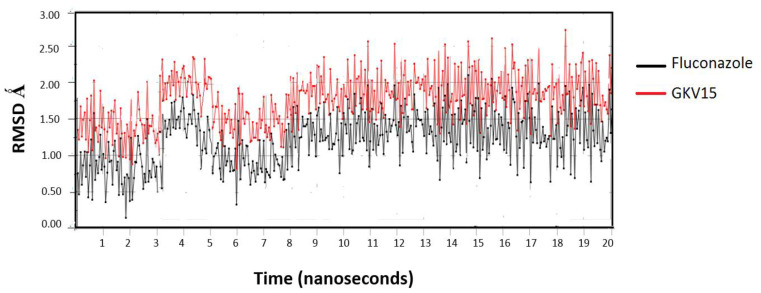
The molecular dynamics simulations over 20 ns.

The top-ranked compound, GKV15, with a high docking score and the best biological result was subjected to a molecular dynamics study against the standard antifungal agent, fluconazole, over 20 nanoseconds (ns) to evaluate the strength of their binding. The oscillations of the tested compound started with RMSD 1.5 Ǻ during the first 3 ns. Then, from 3 ns to 8 ns, there were oscillations suspended between 1.25 Ǻ and 2.00 Ǻ. Equilibrium was reached at 2.00 Ǻ at exactly 8 ns, which was then maintained (Figure 8). Interestingly, these oscillations were parallel to those of fluconazole, but the latter showed lower RMSD values as it reached its equilibrium at 1.5 Ǻ.

## 3. Materials and Methods

### 3.1. General

The synthesis of the test compounds was achieved by two-step chemical synthesis and is depicted in Scheme 1, as per the previous report [36]. The molecular structures are represented in Figure 1. A single-crystal X-ray diffraction study was performed using a Bruker Kappa APEX II DUO diffractometer equipped with a charge-coupled device (CCD) detector, and monochromated Mo K*α* radiation (λ = 0.71073 Å). Data collection was carried out at 173(2) K using an Oxford Cryostream cooling system featuring the Bruker Apex II software. The in vitro antifungal test was performed at the Regional Center for Mycology and Biotechnology, Al-Azhar University, Cairo, Egypt.

### 3.2. Single-Crystal X-Ray Data Collection and Refinement Details

Single-crystal data were collected on a Bruker APEX II diffractometer equipped with a CCD detector, using monochromated Mo K*α* radiation (λ = 0.71073 Å). Unit cell measurement, data collection, integration, scaling, and absorption corrections for the crystal were carried out using the Bruker Apex II software [38]. Data reductions were performed with the Bruker SAINT suite [39]. The crystal structures were solved by direct methods using SIR 92 [40] and refined by the full-matrix least-squares method using SHELXL 2014 [41], which is included in the program suite WinGX (version 2014.1) [42]. Absorption corrections were applied using SADABS [43]. All non-hydrogen atoms were refined anisotropically, and all hydrogen atoms were positioned geometrically and refined using a riding model with Uiso(H) = 1.2Ueq. The Oak Ridge Thermal Ellipsoid Plot (ORTEP) and crystal packing diagrams were generated using the Mercury 3.5.1 (CCDC) program [44]. Geometrical calculations were performed using PARST [45] and PLATON [46]. The single-crystal data collection and refinement details are tabulated in Table 3. Intermolecular interactions involved in the title compound (5-(4-ethylphenyl)-2-methyl-2*H*-1,2,3-triazol-4-yl)(thiophen-2-yl)methanone (GKV10) are summarised in Table 4. Crystal information file (CIF) of compound GKV10 was deposited in The Cambridge Crystallographic Data Centre (CCDC) [47] with CCDC number 1443666 and available as electronic Supplementary Material.

### 3.3. Antifungal Screening

The calculation of the minimum inhibitory concentrations (MICs) for the tested compounds was done against *Candida parapsilosis* (ATCC RCMB 05064); *Candida albicans* (ATCC RCMB 05064); *Candida tropicalis* (ATCC RCMB 05064); *Aspergillus niger* (ATCC RCMB 05064); *Trichophyton rubrum* (ATCC RCMB 05064). Microdilution assay was used to evaluate the tested compounds (GKV1-15) for their antifungal activity [48]. Different concentrations were tested from each compound; 0.49, 0.98, 1.95, 3.9, 7.81, 15.63, 31.25, 62.5, and 125 µg/mL over 48 h. The experiments were done in The Regional Center for Mycology and Biotechnology, Al-Azhar University, Cairo, Egypt. Fluconazole was used as a reference drug.

### 3.4. Molecular Docking

The Molecular Operating Environment (MOE) program was used for performing the docking studies. The chain A was selected as the main chain, which was complexed with heme–porphyrin and a reported azole inhibitor. The amino acids within a radius of 6.5 Å were selected in the binding site. The triangle method was the main placement method. London dG was used as a rescoring method. The Merck molecular force field 94x (MMFF94x) was selected as a force field [49].

### 3.5. Molecular Dynamics

The docking of GKV15 revealed a stable pose that was kept in the active site. The protein geometries, electron density and temperature-related factors were tested. All hydrogens were added, and energy minimization was calculated. Any foreign solvent molecules in the system were deleted; salt atoms were then added to the system to surround the biomolecular protein–ligand complex in a spherical shape. Sodium chloride was added in a 0.1 mol/L concentration. The cell dimensions were of 81.9 × 81.9 × 81.9 Å. The total number of molecules within the system was 18,295, 1.01 g/cm^3^. Assisted Model Building with Energy Refinement 10:Extended Hückel Theory (Amber 10:EHT) was selected as a force field. The heat was adjusted in order to increase the temperature of the system from 0–300 K, which was followed by equilibration and production for 300 ps; cooling was then initiated until 0 K was reached. The simulation was conducted over a 20 ns period of time (20,000 ps).

## 4. Conclusions

A series of 2,4,5-trisubstituted-1,2,3-triazole derivatives (GKV1-15) bearing a substituted phenyl moiety at the fourth position of the 1,2,3-triazole nucleus was synthesized, and the derivatives were evaluated for their antifungal activity. The single-crystal X-ray diffraction studies of compound GKV10 established that C-H···O, C-H···N, C-H···S, C-H···π, and π···π intermolecular interactions primarily stabilized the supramolecular packing arrangement of the molecules in the solid-state. The antifungal results indicate that all the test compounds exhibited promising antifungal effects against the tested ATCC strains. The title compound GKV15 was highly promising, as it exhibited the best antifungal activity against *C. parapsilosis*, *C. albicans*, *C. tropicalis*, *A. niger*, and *T. rubrum* at an MIC of 0.98, 0.98, 0.49, 0.98, and 0.98 µg/mL, respectively. This result was combined with the molecular docking results of free energy, affinity, clash score, and RMSD at −24.75, 30.01, 2.25, and 0.81, respectively. The activity of the test compound GKV15 was similar in magnitude to the standard drug. Thus, the test compound GKV15, reported herein, is a promising starting point for the development of an antifungal drug candidate.

## Figures and Tables

**Table 1:** The antifungal activity of 5-(substituted phenyl)-2-methyl-2*H*-1,2,3-triazol-4-yl(4-substitutedphenyl/thiophenyl) methanone analogues (GKV1-15).

Compound Code	Minimum Inhibitory Concentration (μg/mL)				
	*C. parapsilosis*	*C. albicans*	*C. tropicalis*	*A. niger*	*T. rubrum*
**GKV1**	125	62.5	62.5	125	125
**GKV2**	31.25	15.63	15.63	62.5	62.5
**GKV3**	125	62.5	62.5	125	125
**GKV4**	15.63	15.63	15.63	31.25	31.25
**GKV5**	125	62.5	62.5	125	125
**GKV6**	31.5	15.63	15.63	31.25	31.25
**GKV7**	7.81	7.81	3.9	15.63	15.63
**GKV8**	7.81	7.81	3.9	15.63	15.63
**GKV9**	3.9	3.9	1.98	7.81	15.63
**GKV10**	0.98	0.98	0.49	1.95	1.95
**GKV11**	0.98	0.98	0.49	0.98	1.95
**GKV12**	15.63	15.63	15.63	31.25	31.25
**GKV13**	15.63	15.63	7.81	15.63	15.63
**GKV14**	7.81	7.81	3.9	7.81	15.63
**GKV15**	0.98	0.98	0.49	0.98	0.98
**Fluconazole**	0.49	0.49	0.98	0.49	1.95

*Candida parapsilosis* (ATCC RCMB 05064); *Candida albicans* (ATCC RCMB 05064); *Candida tropicalis* (ATCC RCMB 05064); *Aspergillus niger* (ATCC RCMB 05064); *Trichophyton rubrum* (ATCC RCMB 05064).

**Table 2:** Molecular docking results of the (5-(substituted phenyl)-2-methyl-2*H*-1,2,3-triazol-4-yl)(4-substitutedphenyl/thiophenyl) methanone analogues of GVK1–15.

Compound	Computational Parameters			
	ΔG(kcal/mol)	Affinity	Clash Score	RMSD (Ǻ)
**GKV1**	−22.64	25.88	3.50	1.11
**GKV2**	−22.41	25.72	3.01	1.25
**GKV3**	−22.94	26.32	2.95	1.03
**GKV4**	−22.33	26.10	3.00	1.30
**GKV5**	−22.04	26.34	2.84	1.25
**GKV6**	−22.83	24.97	2.65	1.25
**GKV7**	−23.98	29.45	2.33	1.01
**GKV8**	−23.88	29.33	2.38	0.97
**GKV9**	−23.71	28.95	2.35	0.94
**GKV10**	−24.33	29.80	2.40	0.87
**GKV11**	−24.54	29.87	2.51	0.85
**GKV12**	−21.95	25.12	3.25	1.50
**GKV13**	−21.67	26.74	3.13	1.61
**GKV14**	−22.89	25.74	2.81	1.44
**GKV15**	−24.75	30.01	2.25	0.81

RMSD: root of the mean square deviation.

**Table 3:** Single-crystal data collection and refinement.

Data	Compound GKV10
CCDC	1443666
Formula	C16 H15 N3 O S
Formula weight	297.37
Temperature/K	110 (2)
Wavelength (Å)	0.71073
Solvent system, Temperature	Toluene, 25 °C
Crystal system	Triclinic
Space group	*P*-1
*a* (Å)	7.6676 (4)
*b* (Å)	9.8450 (5)
*c* (Å)	10.8781 (6)
*α* (°)	94.319 (3)
*β* (°)	109.220 (3)
*γ* (°)	107.332 (3)
V (Å^3^)	726.36 (7)
*Z*ʹ, Z	1, 2
Density (g cm^−3^)	1.360
*μ* (mm^−1^)	0.225
F (000)	312
θ (min, max)	2.21, 30.02
h_min, max_, k_min, max_, l_min, max_	−10 10, −13 12, −14 14
No. of ref.	10812
No. of unique ref./obs. Ref.	3573/3098
No. parameters	172
R_all_, R_obs_	0.0503, 0.0442
wR2_all_, wR2_obs_	0.1301, 0.1249
∆ρ_min, max_ (eÅ^−3^)	−0.249, 0.446
Goodness of Fit (G. O. F.)	1.066

**Table 4:** Intermolecular interactions in GKV10: Cg1 refers to the center of gravity of the ring formed by C1-C2-C3-S1-C13.

Motifs	D–H···A	Symmetry	Geometry		
			D···A/Å	H···A/Å	˂D–H···A/°
I	C1-H1···O1	x + 1, +y, +z	3.235 (2)	2.18	164
II	C11-H5···N3	x + 1, +y, +z	3.750 (3)	2.71	161
III	C13 -H3···N3	x + 1, +y + 1, +z	3.430 (2)	2.35	175
IV	C16 -H14···O1	−x, −y, −z + 1	3.546 (2)	2.51	159
V	C16 -H13···N1	−x + 1, −y, −z + 1	3.824 (3)	2.76	167
VI	C2-H4···C14(π)	x + 1, y, z	3.875 (2)	2.99	154
VII	Cg1···Cg1	−x + 1, −y+1, −z + 1	3.901 (2)	−	−
VIII	C9-H11···Cg1	−x + 1, −y + 1,−z + 2	3.911 (2)	3.07	149
IX	C16-H15···S1	x, y − 1, z	3.790 (1)	3.08	131

## Supplementary Materials

The following are available online at https://www.mdpi.com/2079-6382/9/6/350/s1; Supplementary data (Single crystal X-ray CIF file) for compound 5-(4-ethylphenyl)-2-methyl-2*H*-1,2,3-triazol-4-yl(thiophen-2-yl)methanone (GKV10) related to this article can be found as an attachment.
