# Supplementary material for: Crystallography, in Silico Studies, and In Vitro Antifungal Studies of 2,4,5 Trisubstituted 1,2,3-Triazole Analogues
Source: Antibiotics (Basel). 2020 Jun 20;9(6):350. doi: 10.3390/antibiotics9060350 (PMC7344790; doi:10.3390/antibiotics9060350)
Supplement: Supplementary file 1 [file antibiotics-09-00350-s001.zip › Supplementary material/checkCIF_GKV10.pdf]

checkCIF (basic structural check) running

Checking for embedded fcf data in CIF ...
Found embedded fcf data in CIF. Extracting fcf data from uploaded CIF, please wait **.**

checkCIF/PLATON (basic structural check)

Structure factors have been supplied for datablock(s) shelx

THIS REPORT IS FOR GUIDANCE ONLY. IF USED AS PART OF A REVIEW PROCEDURE FOR PUBLICATION, IT SHOULD NOT REPLACE THE EXPERTISE OF AN EXPERIENCED CRYSTALLOGRAPHIC REFEREE.

```
No syntax errors found.                          CIF dictionary
Please wait while processing ....                Interpreting this report
```

Structure factor report

Datablock: shelx

```
Bond precision:      C-C = 0.0021 A              Wavelength=0.71073
Cell:        a=7.6676(4)      b=9.8450(5)      c=10.8781(6)
             alpha=94.319(3)  beta=109.220(3)  gamma=107.332(3)
Temperature:110 K
                   Calculated                      Reported
Volume             726.36(7)                       726.36(7)
Space group        P -1                            P -1
Hall group         -P 1                            -P 1
Moiety formula     C16 H15 N3 O S                  ?
Sum formula        C16 H15 N3 O S                  C16 H15 N3 O S
Mr                 297.37                          297.37
Dx,g cm-3          1.360                           1.360
Z                  2                               2
Mu (mm-1)          0.225                           0.225
F000               312.0                           312.0
F000'              312.35
h,k,lmax           10,13,14                        10,13,14
Nref               3608                            3573
Tmin,Tmax          0.953,0.973                     0.669,0.746
Tmin'              0.935
Correction method= # Reported T Limits: Tmin=0.669
Tmax=0.746 AbsCorr = MULTI-SCAN
Data completeness= 0.990        Theta(max)= 28.279
R(reflections)= 0.0442( 3098)       wR2(reflections)= 0.1301( 3573)
S = 1.066              Npar= 192
```

```
The following ALERTS were generated. Each ALERT has the format
       test-name_ALERT_alert-type_alert-level.
Click on the hyperlinks for more details of the test.
```

Alert level C

```
PLAT911_ALERT_3_C Missing FCF Refl Between Thmin & STh/L=    0.600          13 Report
PLAT913_ALERT_3_C Missing # of Very Strong Reflections in FCF ....           4 Note
```

Alert level G

```
PLAT154_ALERT_1_G The s.u.'s on the Cell Angles are Equal ..(Note)      0.003 Degree
PLAT883_ALERT_1_G No Info/Value for _atom_sites_solution_primary .     Please Do !
PLAT912_ALERT_4_G Missing # of FCF Reflections Above STh/L=  0.600          23 Note
PLAT978_ALERT_2_G Number C-C Bonds with Positive Residual Density.          12 Info
PLAT992_ALERT_5_G Repd & Actual _reflns_number_gt Values Differ by           1 Check
```

```
   0 ALERT level A = Most likely a serious problem - resolve or explain
   0 ALERT level B = A potentially serious problem, consider carefully
   2 ALERT level C = Check. Ensure it is not caused by an omission or oversight
   5 ALERT level G = General information/check it is not something unexpected

   2 ALERT type 1 CIF construction/syntax error, inconsistent or missing data
   1 ALERT type 2 Indicator that the structure model may be wrong or deficient
   2 ALERT type 3 Indicator that the structure quality may be low
   1 ALERT type 4 Improvement, methodology, query or suggestion
   1 ALERT type 5 Informative message, check
```

It is advisable to attempt to resolve as many as possible of the alerts in all categories. Often the minor alerts point to easily fixed oversights, errors and omissions in your CIF or refinement strategy, so attention to these fine details can be worthwhile. In order to resolve some of the more serious problems it may be necessary to carry out additional measurements or structure refinements. However, the purpose of your study may justify the reported deviations and the more serious of these should normally be commented upon in the discussion or experimental section of a paper or in the "special_details" fields of the CIF. checkCIF was carefully designed to identify outliers and unusual parameters, but every test has its limitations and alerts that are not important in a particular case may appear. Conversely, the absence of alerts does not guarantee there are no aspects of the results needing attention. It is up to the individual to critically assess their own results and, if necessary, seek expert advice.

Publication of your CIF in IUCr journals

A basic structural check has been run on your CIF. These basic checks will be run on all CIFs submitted for publication in IUCr journals (*Acta Crystallographica*, *Journal of Applied Crystallography*, *Journal of Synchrotron Radiation*); however, if you intend to submit to *Acta Crystallographica Section C* or *E* or *IUCrData*, you should make sure that full publication checks are run on the final version of your CIF prior to submission.

Publication of your CIF in other journals

Please refer to the *Notes for Authors* of the relevant journal for any special instructions relating to CIF submission.

PLATON version of 22/12/2019; check.def file version of 13/12/2019

Datablock shelx - ellipsoid plot

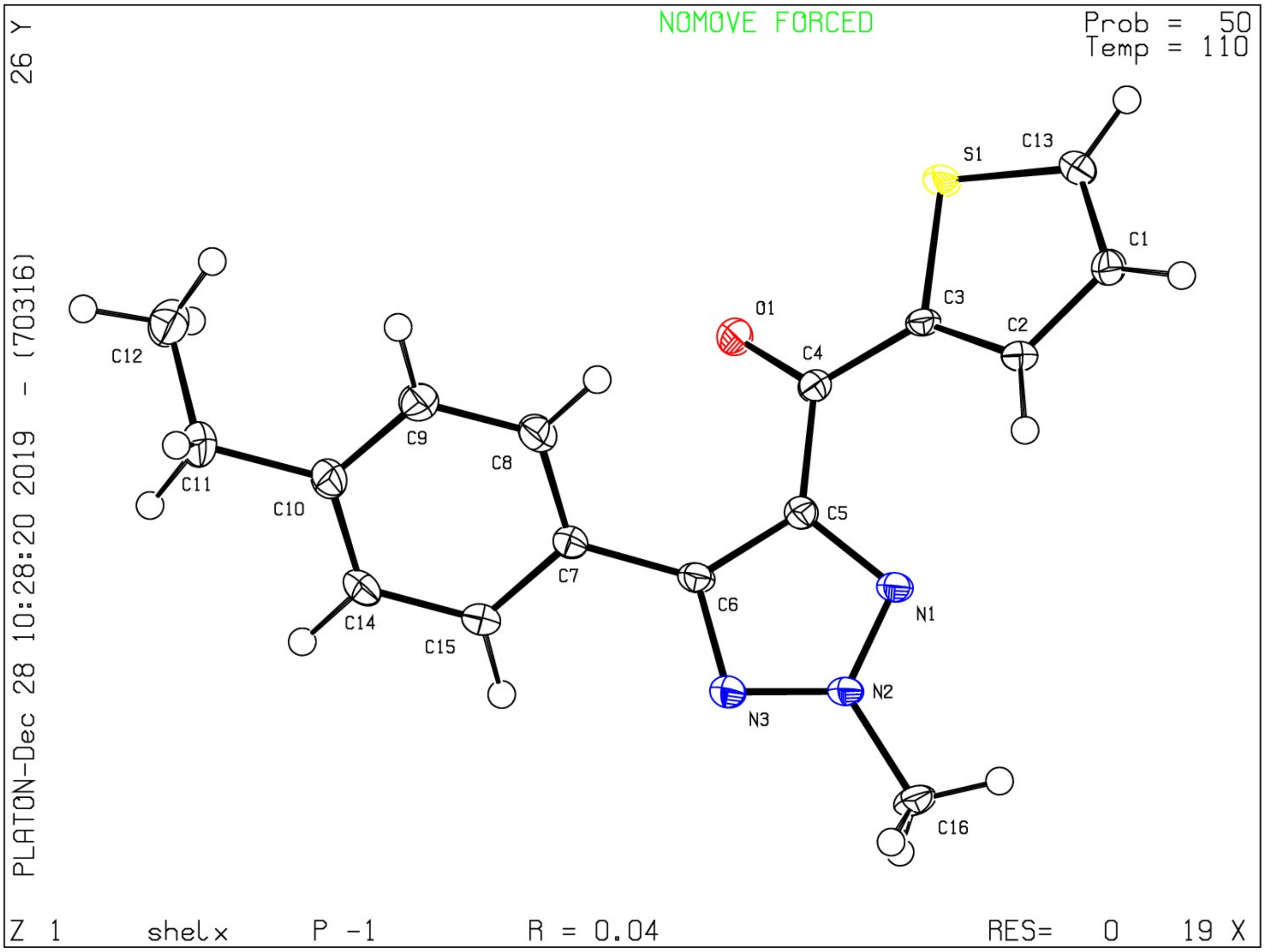

[Download CIF editor (publCIF) from the IUCr](https://checkcif.iucr.org)
[Download CIF editor (enCIFer) from the CCDC](https://checkcif.iucr.org)
[Test a new CIF entry](https://checkcif.iucr.org)
